# Early diagnosis of pancreatic cancer: Current trends and concerns

**DOI:** 10.1002/ags3.12004

**Published:** 2017-04-25

**Authors:** Keiji Hanada, Hironobu Amano, Tomoyuki Abe

**Affiliations:** ^1^ Department of Gastroenterology Onomichi General Hospital Hiroshima Japan; ^2^ Department of Surgery Onomichi General Hospital Hiroshima Japan

**Keywords:** endoscopic ultrasonography (EUS), magnetic resonance cholangiopancreatography (MRCP), pancreatic cancer, regional network, risk factor

## Abstract

Early detection of pancreatic cancer (PC) is essential for a better prognosis. Some recent studies have demonstrated that a slight dilatation of the main pancreatic duct (MPD) and small cystic lesions were detected initially in most cases diagnosed at an early stage. Detecting these abnormal findings in cases with high risk factors through an effective screening system including image diagnosis, some biological markers, or familial cancer registrations should contribute to early diagnosis of PC. It has been reported that endoscopic ultrasonography (EUS) is essential for detecting tumors <10 mm with a favorable prognosis. Additionally, EUS‐guided fine‐needle aspiration biopsy is useful for confirming final histological diagnosis. For the diagnosis of stage 0 PC, local irregular stenosis of MPD should be an important initial abnormal sign detected by EUS or magnetic resonance cholangiopancreatography. Cytodiagnosis multiple times using pancreatic juice obtained by endoscopic nasopancreatic drainage should be essential for the final diagnosis. Recently, activities of regional networks between specialist doctors in medical centers and general practitioners for early diagnosis of PC have been reported in Japan. In the future, these activities may play an important role in the early diagnosis of PC.

## 1 Introduction

Many patients with pancreatic cancer (PC) with a poor prognosis are diagnosed at an advanced stage. This is attributed to the difficulty of early diagnosis of PC.^1^, ^2^ According to the recent Japan Pancreatic Cancer Registry (JPCR) analyzed by Japan Pancreas Society (JPS), the 5‐year survival rate of cases with tumors ≤10 mm (TS1a) reached 80.4%, and the 5‐year survival rate of cases with Stage 0 reached 85.8%.^3^ These data suggest that early diagnosis should play an important role in improving the prognosis of PC. In this manuscript, we would like to review the current trends and concerns of early diagnosis of PC.

## 2 Opportunity to diagnose ‘early pancreatic cancer’

**Figure 1:**
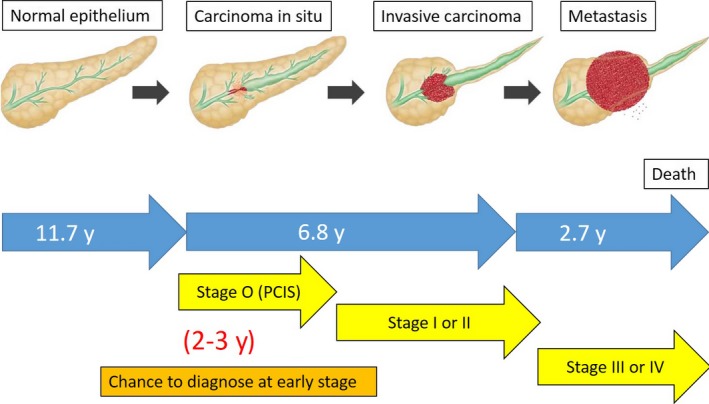
Progression model and stage of pancreatic cancer

Hruban *et al*. first reported a genetic progression model from the precursor lesions named pancreatic intraepithelial neoplasia (PanIN) to PC.^4^ According to their progressions, the extent of atypia was classified as PanIN‐1 (low‐grade dysplasia), PanIn‐2 (moderate‐grade dysplasia), and PanIN‐3 (high‐ grade dysplasia). Cases with PC in situ (PCIS) are classified into PanIN‐3. It has been reported that *K‐ras* mutations in PanIN‐1, *p16* inactivating mutations in PanIN‐2, *TP53* and *SMAD4* inactivating mutations in PanIN‐3 are frequently found. These observations should support a genetic progression model of pancreatic carcinogenesis leading to invasive cancer.^5^, ^6^, ^7^, ^8^, ^9^, ^10^ Recently, the estimated lifetime of clonal evolution during PC development and progression based on a computational model using many autopsy cases has been reported.^11^, ^12^ This model suggested an average of 11.7 years from the initiating carcinogenesis until development of the parental clone, an average of 6.8 years to the development of metastatic subclones within the primary PC, and an average of 2.7 years until death of the case (Figure 1). Most cases with PC were diagnosed toward the end of this lifetime span, suggesting that the poor prognosis was a result of late diagnosis in the natural history of PC. These results suggest that we should have a golden opportunity of 2 or 3 years to diagnose ‘early pancreatic cancer’ including Stage 0 or I.

## 3 TS1a as ‘early pancreatic cancer’

According to the recent JPCR, in cases with TS1a tumor, 65% of them had Union for International Cancer Control (UICC) Stage IA disease. The 5‐year survival of cases with Stage IA reached 68.7%. When the tumor is TS1a, the survival rate was significantly higher than that of cases with tumor >10 mm (≥TS1b).^3^

Recently, Haeno *et al*. investigated PC progression within a mathematical framework of metastasis formation in comprehensive data of 228 cases with PC including 101 autopsy cases. This model revealed that all cases are expected to harbor metastasis‐enabled cells in the primary tumor at the time of diagnosis. Interestingly, a case with the primary tumor of 10 mm has a probability of 28% of harboring metastases at diagnosis; as the primary tumor size increases to 20 mm and 30 mm, the risk of harboring metastases increases to 73 and 94%, respectively.^13^ These results suggest that PC of ≤10 mm with a low potential of metastasis and a favorable prognosis may be defined as ‘early PC’.

## 4 Risk factors and early diagnosis

### 4.1 Intraductal papillary mucinous neoplasm and cystic lesions

In 2013, JPS published clinical guidelines (CGL) for PC based on evidence‐based medicine.^14^ This CGL provides an algorithm for the diagnosis and treatment of PC including 35 clinical questions and 57 recommendations. In clinical question 1, some risk factors have been suggested for the developing PC (Table 1). Patients with more than one risk factor are recommended to carry out further examinations to detect PC. There have been some reports on the development of PC during follow up of cases with branch duct intraductal papillary mucinous neoplasm (IPMN) or pancreatic cysts.^15^, ^16^ Additionally, there have been some retrospective studies of PC concomitant with IPMN (Table 2).^17^, ^18^, ^19^, ^20^, ^21^, ^22^, ^23^, ^24^, ^25^, ^26^, ^27^, ^28^, ^29^, ^30^ These studies demonstrated that the frequency of PC concomitant with IPMN ranged from 1.1% to 11.2%. As for branch duct IPMN, two working groups of JPS reported that seven PC cases were detected in 349 branch duct IPMN cases during the follow‐up period,^17^ and that PC concomitant with IPMN may be diagnosed earlier than ordinary PC.^18^ These observations suggest that patients with IPMN or pancreatic cysts should be carefully observed as having premalignant disease of PC.

**Table 1:** Risk factors for pancreatic cancer (from Clinical Guidelines for Pancreatic Cancer 2013)

Family history	Pancreatic cancer
	Hereditary pancreatic cancer syndrome
Accompanying diseases	Diabetes mellitus
	Obesity
	Chronic pancreatitis
	Hereditary pancreatitis
	Intraductal papillary mucinous neoplasm
	Pancreatic cysts
Habits	Tobacco use
	Heavy drinking

**Table 2:** Previous reports of pancreatic cancer concomitant with IPMN

Author (Year)	No. cases with IPMN	PC concomitant with IPMN	Frequency (%)	Follow‐up period (years)
Yamaguchi *et al*. (2002)^19^	76	7	9.2	no data
Tada *et al*. (2006)^16^	197^a^	5	2.6	3.8
Hanada *et al*. (2006)^20^	60	2	3.3	2
Uehara *et al*. (2008)^15^	60	5	8	7.3
Ingakul *et al*. (2010)^21^	236	22	9.3	no data
Tanno *et al*. (2010)^22^	168	9	5.4	no data
Ikeuchi *et al*. (2010)^23^	145	5	3.4	4.6
Kanno *et al*. (2010)^24^	159	7	4.4	no data
Sawai *et al*. (2010)^25^	103	2	1.9	4.9
Kawakubo *et al*. (2011)^26^	642	17	2.6	4.8
Maguchi *et al*. (2011)^17^	349	7	2	3.7
Yamaguchi *et al*. (2011)^18^	765	31	4.1	no data
Ohno *et al*. (2011)^27^	142	2	1.4	3.5
Ohtsuka *et al*. (2013)^28^	179	20	11.2	no data
Kamata *et al*. (2014)^29^	167	18	10.8	3.5
Crippa *et al*. (2017)^30^	281	3	1.1	4.3

a No. cases included intraductal papillary mucinous neoplasm (IPMN) and cystic regions.

### 4.2 Family history and hereditary PC syndrome

According to the registry of the National Familial Pancreas Tumor Registry (NFPTR), the risk of PC was 6.8‐fold higher in the relatives of cases with familial PC, and 2.4‐fold higher in relatives of cases with sporadic PC.^31^ It has been reported that BRCA2, PALB2, and ataxia telangiectasia mutated germ‐line mutations are most frequently identified in familial PC cases.^32^, ^33^, ^34^ In July 2013 in Japan, JPS established the familial PC registry for early diagnosis, and already started this registry in 2015.

Recently, several genetic risk factors and syndromes have been associated with an increased risk of PC. Hereditary pancreatitis (SPINK1 mutations), hereditary breast ovarian syndrome (BRCA1 and BRCA2 mutations), Peutz‐Jeghers syndrome (STK11/LKB1 mutations), familial atypical multiple mole syndrome (CDKN2A mutations), Lynch syndrome (defects in MLH1, MSH2, MSH6, or PMS2), and familial adenomatous polyposis (APC mutations) have been associated with an increased risk of PC.^32^, ^35^, ^36^, ^37^, ^38^, ^39^ Identification of these cases should allow for focused screening in high‐risk populations. Several initial screening studies using magnetic resonance imaging (MRI) or endoscopic ultrasonography (EUS) have been carried out in cases with genetic risk factors and syndromes, demonstrating some initial potential in identifying PC and premalignant lesions with malignant potential.^40^, ^41^, ^42^ In 2016, these hereditary diseases will be newly added to risk factors for PC in the revised CGL issued by JPS Working Group (Table 3).

**Table 3:** Risk factors for pancreatic cancer (from Clinical Guidelines for Pancreatic Cancer 2016)

Family history	Pancreatic cancer
	Familial pancreatic cancer
Hereditary diseases	Hereditary pancreatitis
	Hereditary breast ovarian cancer syndrome
	Peutz‐Jeghers syndrome
	Familial atypical multiple mole melanoma
	Lynch syndrome
	Familial adenomatous polyposis
Accompanying diseases	Diabetes mellitus
	Obesity
	Chronic pancreatitis
	Intraductal papillary mucinous neoplasm
	Pancreatic cysts
Habits	Tobacco use
	Heavy drinking
Occupation	Exposures to chlorinated hydrocarbon compounds

## 5 Potential biomarkers for early diagnosis

At present, some serum markers such as CA19‐9, carcinoembryonic antigen (CEA) and DUPAN‐2 have been commonly used. However, these markers are not useful for early diagnosis of PC.^43^, ^44^ Recently, some panels of new potential biomarkers based on biological, immunological, and genetic changes of PC using blood samples or body fluids, such as urine and saliva, have been reported. A serum metabolomics‐based diagnostic model and salivary transcriptomic biomarkers for diagnosis of resectable PC are reported to possess higher accuracy than conventional markers.^45^, ^46^ Fukutake *et al*. reported that the plasma free amino acid (PFAA) profile of PC was significantly different from that of healthy controls, and that the PFAA index was a promising biomarker for screening and diagnosis of PC.^47^ Several studies reported that patterns of micro‐RNAs (miRNAs) from circulating exosomes have shown potential as diagnostic markers in PC. Yu *et al*. detected altered expressions in 35 of 700 miRNAs in pancreatic juice of PanIN‐3 cases using quantitative real‐time polymerase chain reaction.^48^ Kojima *et al*.^49^ reported a diagnostic index using expression profiles of the 10 most significant miRNAs, and that the assessment of these markers would be clinically valuable to identify resectable cases of PC. Recently, Gerdtsson *et al*. reported recombinant antibody microarrays identifying serum protein markers associated with different tumor locations in the pancreas.^50^ As for the endoscopic approach, a minimally invasive and simple screening test for early diagnosis of PC using duodenal juice (DJ) has been reported. The sensitivity of S100P in DJ to diagnose PC was higher than that of serum tumor or cytology using pancreatic juice.^51^

## 6 Screening programs for high‐risk cases

The largest screening program is carried out by Johns Hopkins University and involves 24 American Centers of Excellence (CAPS Study). In a recent CAPS3 study, 216 asymptomatic adult high‐risk cases for PC at five medical centers were screened using computed tomography (CT), MRI, and EUS. This program revealed more than one pancreatic mass or a dilatation of the pancreatic duct in 92 (43%) of these cases. CT, MRI, and EUS detected an abnormal pancreatic finding (pancreatic cysts or a dilated pancreatic duct) in 11%, 33.3%, and 43.6% of the high‐risk cases, respectively. Three cases with high‐grade dysplasia in IPMN or multiple intraepithelial neoplasms were finally diagnosed.^52^ A German study (FaPaCa) enrolled 76 high‐risk cases in a screening program using annual EUS, magnetic resonance cholangiopancreatography (MRCP), and laboratory tests for 5 years. These observations gave a diagnostic yield of 1.3% in detecting PanIN‐3.^53^ These results suggest that screening of high‐risk cases of PC could frequently demonstrate small cystic lesions, including premalignant lesions and non‐invasive PC, and that EUS and MRI may be better than CT for the early diagnosis of PC. However, given the low diagnostic yield and taking into consideration the cost and psychological stress of high‐risk cases, effective and non‐invasive new biomarkers should be established in the near future.

## 7 Algorithms of image diagnosis for early PC

Ultrasonography (US) should be an important first‐step imaging modality. It has been reported that a slight dilatation of the MPD and pancreatic cysts detected by US are important predictive sings. Tanaka *et al*. diagnosed 12 cases of PC including Stages 0 and I of 1058 prospective follow‐up cases with these predictive signs, and recommended periodic checks in cases with these predictive signs.^54^

**Figure 2:**
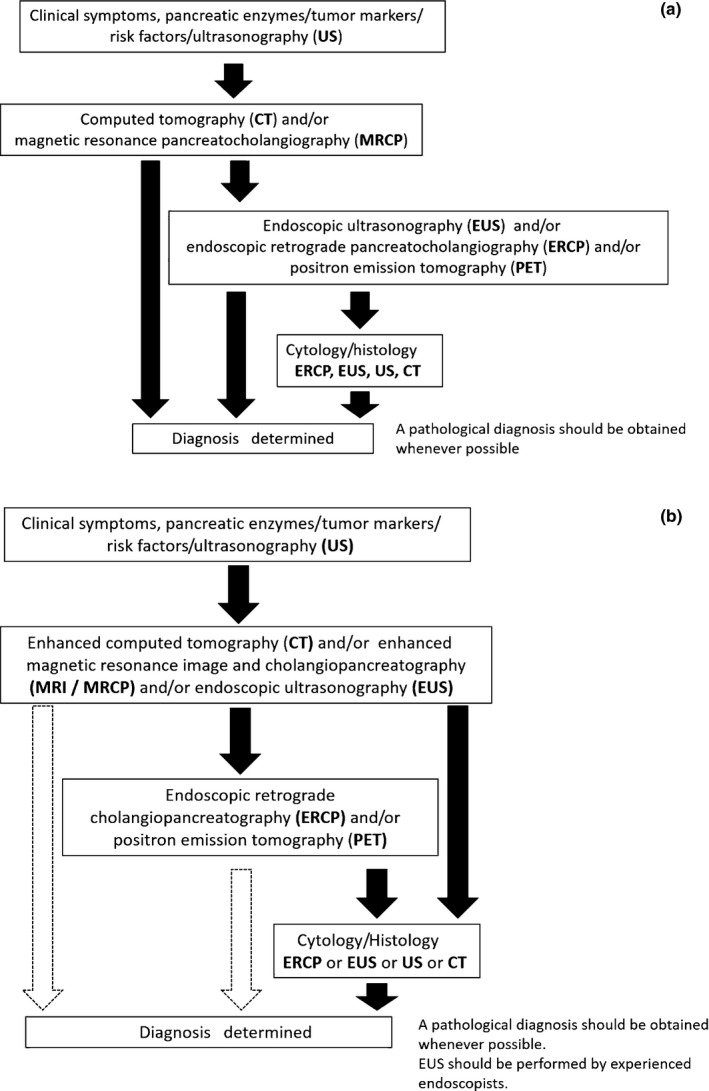
(a) Algorithm for diagnosis of pancreatic cancer (from Clinical Guidelines for Pancreatic Cancer 2013). (b) Algorithm for diagnosis of pancreatic cancer (from Clinical Guidelines for Pancreatic Cancer 2016)

As for image diagnosis using EUS, Yasuda *et al*.^55^ retrospectively examined 132 cases with high‐risk factors for PC without a detectable mass on CT. EUS could detect a small PC <10 mm in three cases. Kamata *et al*. reported the follow‐up data of 102 cases whose branch duct IPMN were followed up using semi‐annual EUS and annual US, CT and MRI. Of these cases, 11 IPMN‐concomitant PC were diagnosed at first examination, and seven IPMN‐concomitant PC were diagnosed during follow‐up periods.^29^ These observations suggest that EUS should have important roles in the early diagnosis of PC. Recently, JPS first published a recommendation for early diagnosis of PC with a favorable long prognosis in the current CGL as follows.^14^ Dilatation of the main pancreatic duct and the presence of cysts are important indirect signs. MRCP and EUS are recommended even when US and CT fail to directly detect a mass lesion. In 2016, the algorithm for diagnosis of PC will be improved in revised CGL issued by JPS Working Group. EUS will be recommended as the second‐step examination for diagnosis of PC (Figure 2).

## 8 Regional networks for early diagnosis of PC (Onomichi project)

**Figure 3:**
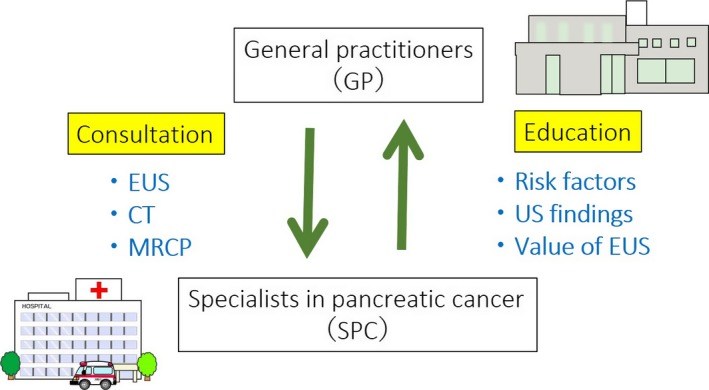
Concept of the regional network for early diagnosis of pancreatic cancer (PC) (Onomichi project). CT, computed tomography; EUS, endoscopic ultrasonography; MRCP, magnetic resonance cholangiopancreatography; US, ultrasonography

**Figure 4:**
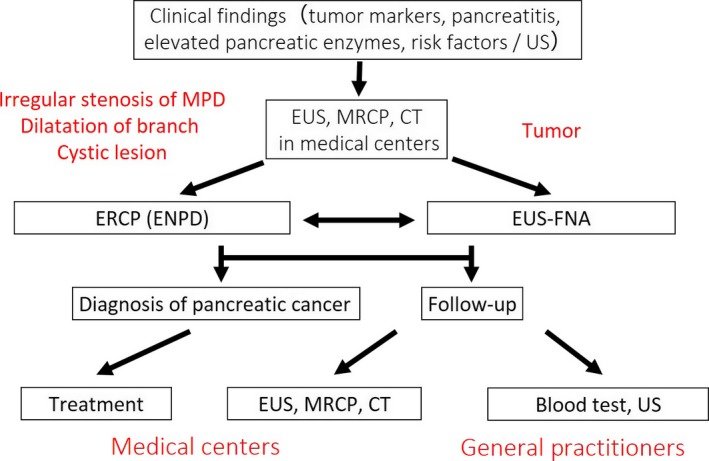
Algorithm of the Onomichi project for early diagnosis of pancreatic cancer (from reference 56). CT, computed tomography; ENPD, endoscopic nasopancreatic drainage; ERCP, endoscopic retrograde cholangiopancreatography; EUS, endoscopic ultrasonography; EUS‐FNA, EUS‐guided fine‐needle aspiration; MPD, main pancreatic duct; MRCP, magnetic resonance cholangiopancreatography; US, ultrasonography

It has been reported that regional networks between specialists in PC (SPC) and general practitioners (GP) should play an important role for early diagnosis of PC. Onomichi city is a rural city located in Hiroshima Prefecture in western Japan, and its total population is approximately 150 000. Onomichi General Hospital and Onomichi Medical Association established a community program for early diagnosis of PC in 2007 (Figure 3). From January 2007 to June 2014, a total of 6475 cases consulted SPC after starting this program. After carrying out CT, MRI, and EUS to detect suspicious findings of PC, such as mass lesions, dilatation of MPD or cystic regions, endoscopic retrograde cholangiopancreatography (ERCP) or EUS‐guided fine‐needle aspiration (EUS‐FNA) was carried out. If irregular stenosis of the MPD was observed on ERCP, cytodiagnosis multiple times using pancreatic juice obtained by endoscopic nasopancreatic drainage (ENPD) was additionally done (Figure 4). As a result, 399 out of 6475 cases were histologically diagnosed as PC. Of these cases, 16 were finally diagnosed as PCIS.^56^ As the concept of the Onomichi project spreads, some Japanese medical associations have tried to establish the regional network for early diagnosis of PC. In the future, regional networks between SPC and GP in medical associations for early diagnosis of PC should be established in other rural areas in Japan.

## 9 Diagnosis of PCIS

According to a recent JPCR by JPS, the 5‐year survival rate of cases with UICC Stage 0 is 85.8%, which is a satisfactory prognosis.^3^ However, it has been difficult to diagnose PCIS without the presence of a formed mass lesion by any imaging modalities. As for the early state of invasive PC, Ikeda *et al*. reported that the non‐invasive cancer parts of invasive PC were classified into three types: flat (F), low papillary (LP) and mixed (FLP). Interestingly, the LP type had a greater tendency than the F type to spread intraductally. The LP type seemed to change to invasive cancer after or while spreading intraductally to some extent, whereas the F type seemed to invade with little intraductal spread.^57^ In cases with pTS1 (histologically ≤2 cm in diameter), the frequency of intraductal spread of PC was high.^58^, ^59^ Understanding these processes of growth and development of PC with an early stage should contribute to the diagnosis of PCIS.

**Figure 5:**
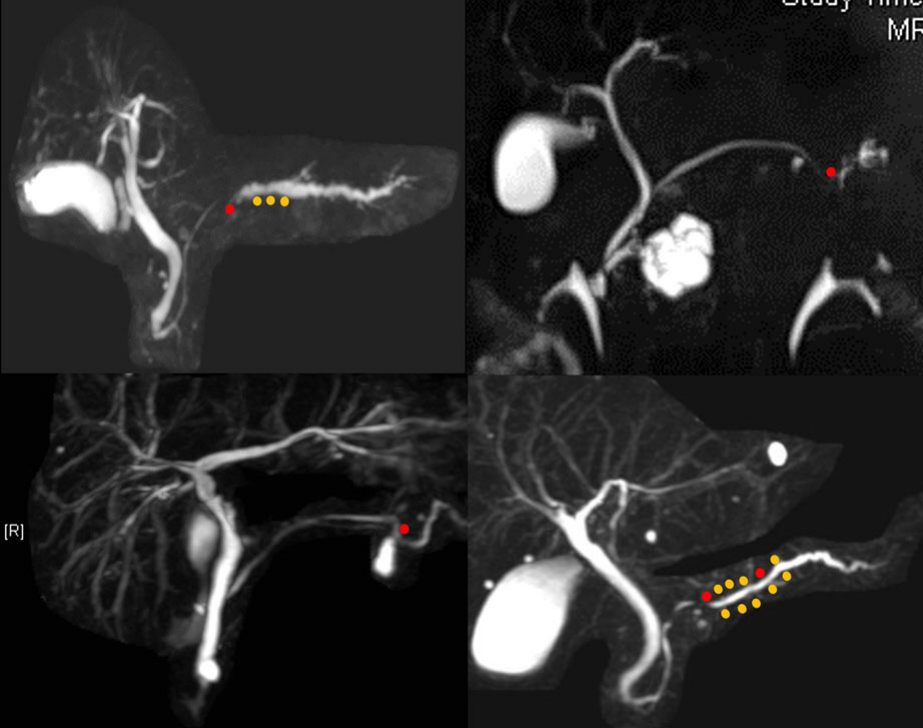
Magnetic resonance cholangiopancreatography findings of pancreatic cancer in situ (PCIS). Localized stenosis with distal dilatation, irregularity, non‐continuous narrowing, focal ductal branch dilatations and cystic lesions around the main pancreatic duct are observed. Red dot, PCIS; yellow dot, atypical epithelium

**Figure 6:**
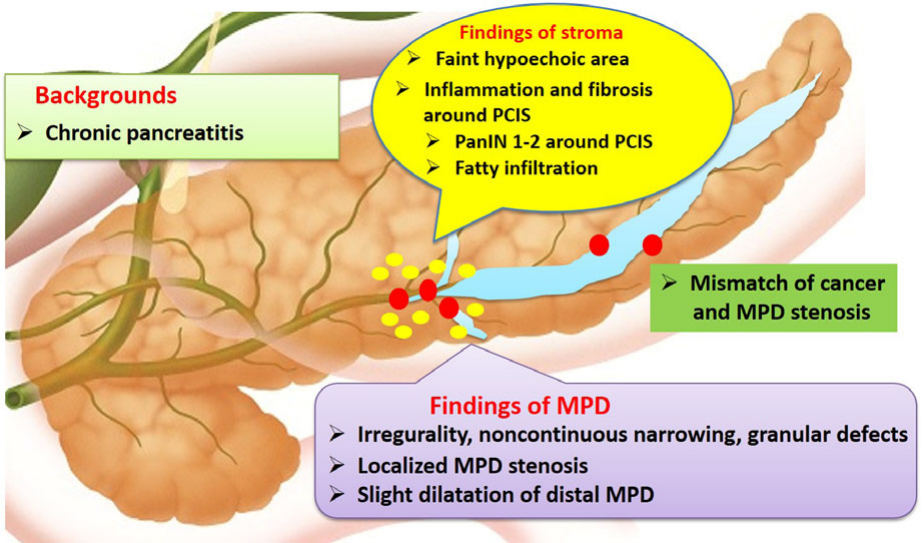
Summary of image and pathological findings of PCIS. MPD, main pancreatic duct; PanIN, pancreatic intraductal neoplasm; PCIS, pancreatic cancer in situ

As for image diagnosis of PCIS, there have been a few reports using various imaging modalities. Abnormal findings in the MPD, such as localized stenosis with distal MPD dilatation, irregularity, non‐continuous narrowing and granular defects were frequently observed by endoscopic retrograde pancreatography (ERP), MRCP or EUS. Focal ductal branch dilatations and cystic lesions around the MPD were also observed (Figure 5).^56^, ^60^, ^61^, ^62^ Recently, Kikuyama *et al*. reported that three out of 14 cases with PCIS had a high degree of fatty changes of the pancreatic parenchyma adjacent to PCIS, which were recognized on CT.^63^ There have been some interesting reports about pathological findings of PCIS using resected specimens. Localized pancreatitis with infiltration of inflammatory cells, fibrosis, and fatty infiltration were frequently observed in the parenchyma around PCIS and atypical epithelium. In addition, there were some PCIS cases with intraductal spread, and mismatch of cancer and MPD stenosis.^56^, ^63^, ^64^, ^65^, ^66^, ^67^, ^68^, ^69^ EUS could detect localized pancreatitis, fibrosis, and fatty infiltration around PCIS as a slightly low echoic lesion.^56^, ^66^ Further examinations will be needed to confirm these possibilities in the future (Figure 6).

For pathological diagnosis of PCIS, cytodiagnosis using pancreatic juice (PJ) during ERCP has been reported to be useful. Recently, there have been some reports of cytodiagnosis multiple times using PJ obtained by ENPD. The sensitivity and accuracy for diagnosis of PCIS using this method was 100%, and 95%, respectively.^64^, ^70^, ^71^ Current Japanese CGL for PC recommends cytodiagnosis multiple times using PJ during ERCP when localized stenosis of MPD is observed by MRCP, EUS, or ERCP.^14^

## Conflicts of interest

Hanada K. has the following financial relationships to disclose: Honoraria (lecture and manuscript fee) from: Eisai Co. Ltd. The other authors declare no conflicts of interest for this article.
